# Structural systems biology: from bacterial to cancer networks

**DOI:** 10.1186/1471-2164-15-S2-O14

**Published:** 2014-04-02

**Authors:** Adam Godzik

**Affiliations:** 1 Bioinformatics and Systems Biology Program, Sanford-Burnham Medical Research Institute, 10901 N. Torrey Pines Rd., La Jolla, CA 92037, USA; 2 Center of Excellence in Genomic Medicine Research, King Abdulaziz University, P.O. Box: 80216 Jeddah 21589, Kingdom of Saudi Arabia

## 

A new research field of systems biology integrates available information about individual components of a biological system to construct a network model which can be analyzed and simulated to predict behavior of cells and organisms. At the same time, structural biology provides information about molecular, three dimensional structure of proteins, which are nodes in this network. However, these two fields historically developed separately and insights from the structures were not used to analyze networks, and vice versa, network information was now used to understand protein structures. We developed integrated, structural and network models of central metabolism in bacterial model systems, T. maritima and E. coli. This allowed us to study first the internal structure and history of the expansion of the network and then attempt quantitative predictions of the network failure in high temperatures. We are now applying similar integrated approach to model network consequences of cancer mutations, including effect of drug sensitivity on specific cancer cell lines.

